# Strategy To Assess Zoonotic Potential Reveals Low Risk Posed by SARS-Related Coronaviruses from Bat and Pangolin

**DOI:** 10.1128/mbio.03285-22

**Published:** 2023-02-14

**Authors:** Yong Yang, Xu-Rui Shen, Yu-Lan Zhang, Ren-di Jiang, Xi Wang, Zhen-Qiong Guan, Qian Li, Yu-Lin Yao, Qian-chun Gong, Rong Geng, Qi Wang, Yan Zhu, Jing-Yi Luo, Zheng-Li Shi, Hui-lan Zhang, Ke Peng, Peng Zhou

**Affiliations:** a CAS Key Laboratory of Special Pathogens and State Key Laboratory of Virology, Center for Antiviral Research, Wuhan Institute of Virology, Chinese Academy of Sciences, Wuhan, Hubei, China; b University of Chinese Academy of Sciences, Beijing, China; c State Key Laboratory of Genetic Engineering, School of Life Sciences, Fudan University, Shanghai, China; d Center for Organoid and Regenerative Medicine, Greater Bay Area Institute of Precision Medicine, Guangzhou, China; e NHC Key Laboratory of Respiratory Diseases, Department of Respiratory and Critical Care Medicine, Tongji Hospital, Tongji Medical College, Huazhong University of Sciences and Technology, Wuhan, China; f Guangzhou Laboratory, Guangzhou International Bio Island, Guangzhou, Guangdong Province, China; University of Hong Kong

**Keywords:** severe acute respiratory syndrome-related coronavirus, zoonosis, risk assessment, bat, pangolin

## Abstract

In the last 2 decades, pathogens originating in animals may have triggered three coronavirus pandemics, including the coronavirus disease 2019 pandemic. Thus, evaluation of the spillover risk of animal severe acute respiratory syndrome (SARS)-related coronavirus (SARSr-CoV) is important in the context of future disease preparedness. However, there is no analytical framework to assess the spillover risk of SARSr-CoVs, which cannot be determined by sequence analysis alone. Here, we established an integrity framework to evaluate the spillover risk of an animal SARSr-CoV by testing how viruses break through key human immune barriers, including viral cell tropism, replication dynamics, interferon signaling, inflammation, and adaptive immune barriers, using human *ex vivo* lung tissues, human airway and nasal organoids, and human lung cells. Using this framework, we showed that the two pre-emergent animal SARSr-CoVs, bat BtCoV-WIV1 and pangolin PCoV-GX, shared similar cell tropism but exhibited less replicative fitness in the human nasal cavity or airway than did SARS-CoV-2. Furthermore, these viruses triggered fewer proinflammatory responses and less cell death, yet showed interferon antagonist activity and the ability to partially escape adaptive immune barriers to SARS-CoV-2. Collectively, these animal viruses did not fully adapt to spread or cause severe diseases, thus causing successful zoonoses in humans. We believe that this experimental framework provides a path to identifying animal coronaviruses with the potential to cause future zoonoses.

## INTRODUCTION

Zoonoses account for the majority of emerging infectious diseases in humans. Indeed, pathogens originating in animals are thought to have triggered most of the viral pandemics that have occurred in the last 2 decades, including the three coronavirus disease pandemics (1). The yearly probability of a pandemic occurring is expected to increase severalfold in the coming decades, owing to human-induced environmental changes which may create opportunities for contact between humans and wild animals (2). In addition, some wild animals carry viruses that may spread to humans. For example, *Rhinolophus* bats naturally carry a large variety of severe acute respiratory syndrome (SARS)-related coronaviruses (SARSr-CoVs), some of which are closely related to SARS-CoV-1 and SARS-CoV-2 (3, 4). Thus, evaluation of the spillover risk of animal viruses is as important as ecological efforts, such as stopping deforestation, in the context of future disease preparedness.

However, there are two main misconceptions regarding the risk of animal viruses: (i) animal viruses can readily infect humans and (ii) human receptor usage may cause spillover. For example, bats carrying SARSr-CoVs are considered a threat to humans. However, only a small percentage of these viruses can utilize the human angiotensin-converting enzyme 2 (ACE2) receptor (3). Furthermore, some bat- and pangolin-derived SARSr-CoVs present a risk of affecting humans owing to their strong binding capacities to human ACE2 and wide infection spectra (5–8). However, spike-receptor binding is only the first step for successful zoonosis, and the key event in zoonosis is when an animal virus begins to replicate in the first human subject via interaction with host proteins in the new host (9). Unfortunately, despite extensive international efforts on genome sequencing, an analytic framework to assess the spillover risk of bat SARSr-CoVs has not yet been established.

We argue that the spillover risk of bat CoVs can be evaluated by testing how viruses break through key human genetic barriers, including receptors, replication, and host defense, in an integrated framework. Here, we established an integrated framework and compared the zoonotic risk of human SARS-CoV-2, bat-derived WIV1-CoV, and pangolin-derived PCoV-GX, three representative human and animal SARSr-CoVs that use the human ACE2 receptor and were isolated in cell culture (4, 7, 8). Our work provides a pathway for identifying animal CoVs with the potential to cause zoonotic events.

## RESULTS

### Strategy to assess zoonotic potential of bat SARSr-CoVs in humans.

**FIG 1:**
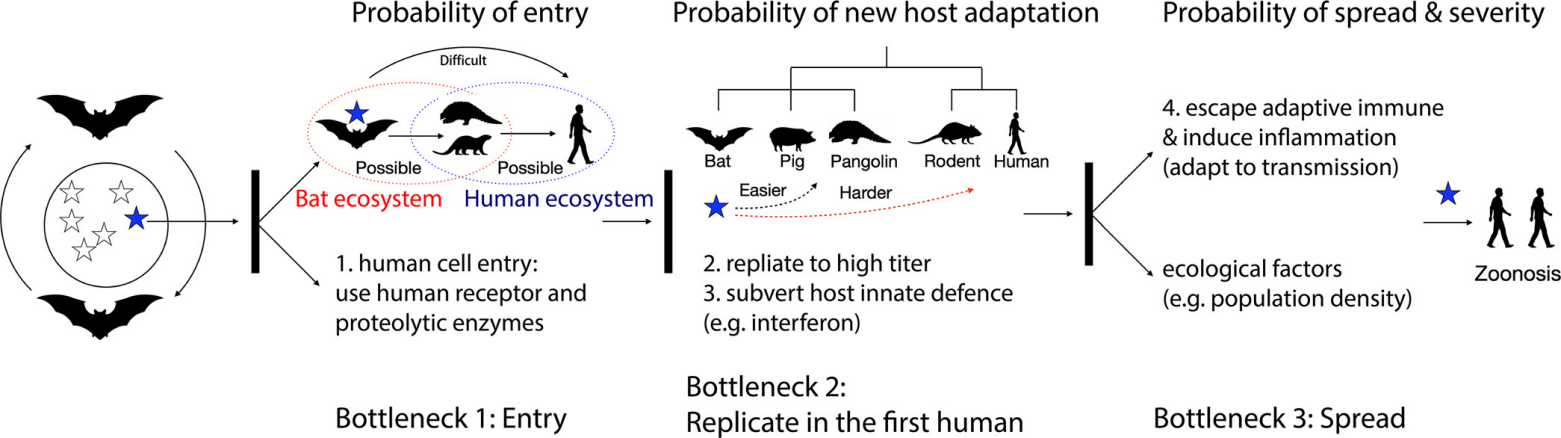
A strategy to assess the zoonotic risk of a bat coronavirus. There are three bottlenecks for a bat coronavirus to break through. Multiple factors, including viral traits, host species characteristics, and ecological factors, should be considered. Only a few bat SARSr-CoVs have the probability of spillover via human cell entry mechanisms and have the opportunity to infect (the more likely new host species would be one that shares the same ecosystem as bats), and fewer may replicate to high titers by subverting host immunity or recruiting host proteins. Notably, it would be easier for a virus to adapt to a new host that is phylogenetically closer to the natural reservoir host (e.g., a virus moving from monkeys to humans). In some cases, a virus may adapt to causing zoonosis in humans.

As natural reservoir hosts, bats harbor a large variety of genetically diverse SARSr-CoVs, and this large quasispecies pool may facilitate the emergence of viruses capable of infecting human cells through recombination (10). However, we believe that zoonotic events caused by direct infection with bat CoVs are extremely rare, because there are at least three bottlenecks (Fig. 1):

1. Probability of spillovers. Only CoVs that use human entry receptors and proteolytic enzymes are capable of infecting human cells (step 1). Thus, it is conceivable that only a very limited percentage of bat SARSr-CoVs may cross this barrier by showing human cell entry or isolation. Opportunity is another key determinant of zoonosis. Thus, it would be easier for a bat virus to infect animals that share the same ecosystem with bats rather than to infect humans (rarely in contact with bats), whereas deforestation and wildlife trading increase the opportunity for human-animal virus contact.
2. Probability of new host adaptation. It is critical for an animal virus to interact with and recruit host proteins needed to replicate to a high titer upon infection of the first human (step 2). Thus, it would be easier for a bat virus to replicate in a phylogenetically closer mammal (e.g., pigs or pangolins) than in rodents or humans. By subverting host defense mechanisms (e.g., interferon [IFN], step 3) and recruiting host proteins necessary for replication, a bat CoV may replicate to a titer required for spread.
3. Probability of spread and severity. Considering wide vaccination efforts and the prevalence of natural SARS-CoV-2 infections, animal SARSr-CoVs that could break these adaptive immune barriers, such as neutralizing antibodies and T-cell immunity, may have the advantage of spreading. Moreover, viruses that induce prominent inflammation or cell death, particularly in the upper respiratory or intestinal tract, may also have advantages against viruses that fail to do so in terms of pathogenesis or spread, because inflammation and cytopathology contribute to increased airway and/or intestinal congestion (e.g., coughing or diarrhea) and viral transmission (step 4) (11). Finally, population density and trafficking are factors that influence viral spread.

Thus, we believe that only bat CoVs that break through all three barriers are dangerous candidates for causing future zoonoses, and most of these frameworks can only be assessed using wet lab experiments.

### SARSr-CoVs from bats and pangolins share the same cell tropism but have replication dynamics different from those of SARS-CoV-2 in the lungs.

**FIG 2:**
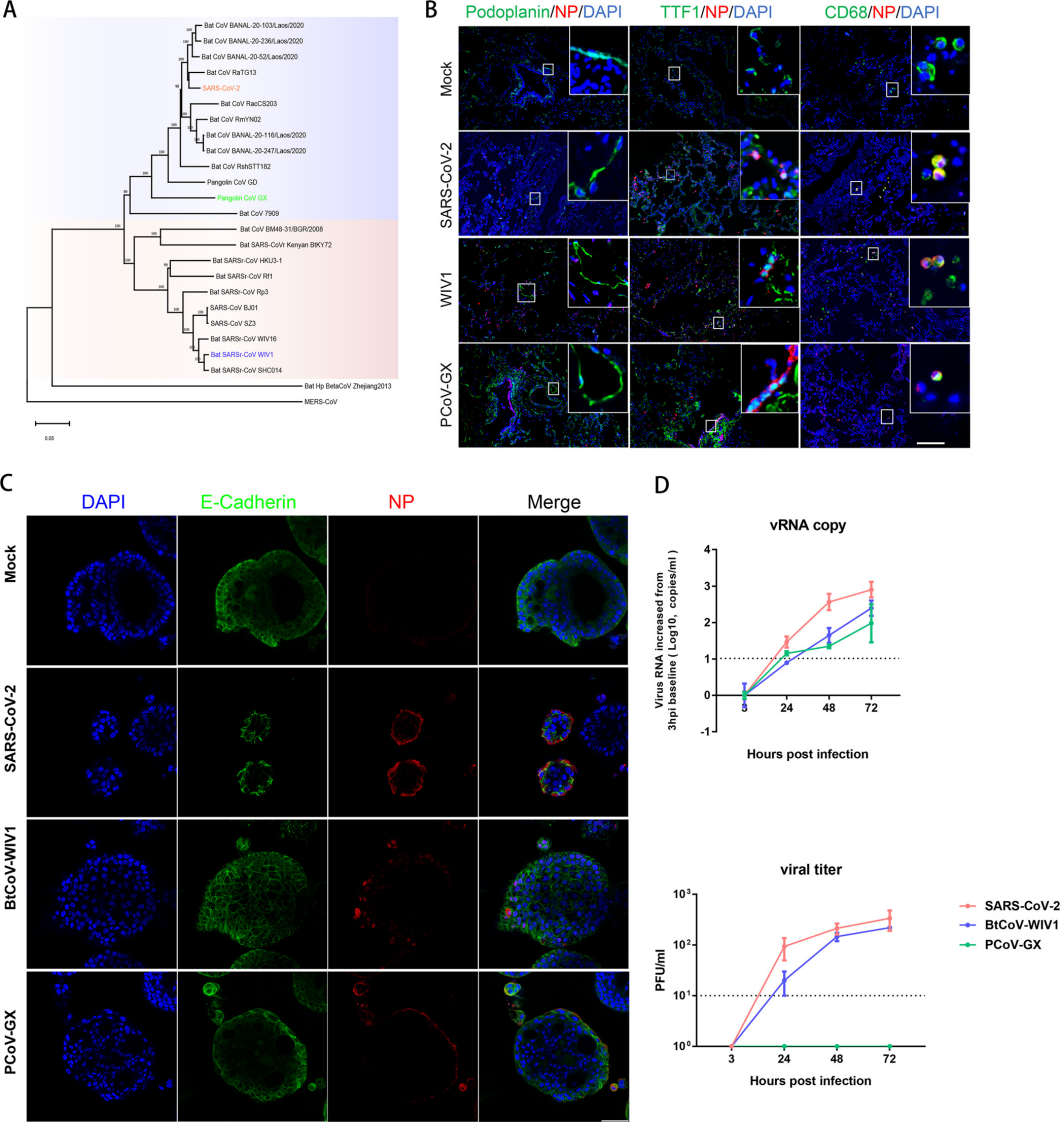
Infection and replication dynamics of human, bat, and pangolin SARSr-CoVs in *ex vivo* human lung tissues and human airway organoids. (A) Phylogeny of SARSr-CoVs. The three viruses tested in the experiments are colored. (B) Cell type spectrum in human lungs. Human lung tissues were infected with SARS-CoV-2, BtCoV-WIV1, or PCoV-GX. At 48 h postinfection, the tissues were fixed in 4% paraformaldehyde and immunohistochemically stained for the indicated cell markers: anti-podoplanin/gap36 for type I pneumocytes (green), anti-TTF1 for type II pneumocytes (green), anti-CD68 (green) for macrophages, anti-SARS-CoV-2 nucleoprotein for virus-infected cells (red), and DAPI (blue) for nuclei. Mock-infected lung tissues were used as controls. Bars, 200 μm. (C and D) Infections in human airway organoids. The same infection conditions were used. The viral NP protein (red), epithelial cell marker E-cadherin (green), and nuclei (blue) are indicated (C). Bars, 50 μm. Culture supernatants were harvested at 3, 24, 48, and 72 h postinfection, and the qPCR-determined viral RNA copies or infectious viral particles (in PFU per milliliter) (D) are shown. The detection limits are indicated.

We then evaluated the zoonotic risk of animal SARSr-CoVs using this framework. Although SARSr-CoVs are widely found in bats and occasionally found in other animals (e.g., pangolins and civets), only a few of have been isolated (3, 8). The isolates included a bat CoV WIV1 (BtCoV-WIV1), which is closely related to human SARS-CoV-1, and a pangolin CoV-GX (PCoV-GX), which is related to human SARS-CoV-2 (Fig. 2A). In previous studies, we demonstrated that both viruses use human ACE2 and proteolytic enzymes for cell entry into cultured human cell lines and thus should break down bottleneck 1 (5, 8, 12).

To determine whether BtCoV-WIV1 and PCoV-GX truly infect human lungs, we first used *ex vivo* human lung tissues, with SARS-CoV-2 as a control. Human lung tissues from five donors who underwent surgery were collected, divided into three parts per tissue, and infected with the three viruses. The results showed that BtCoV-WIV1 and PCoV-GX shared the same cell tropism as SARS-CoV-2 in the lungs. At 48 h postinfection, abundant viral nucleoprotein (NP) antigen positivity was observed in type II pneumocytes (TTF1) and alveolar macrophages (CD68), but not in type I pneumocytes (podoplanin) (Fig. 2B). Viral NP signals were not detected in mock-infected tissue. To quantify the replication profiles of the three viruses in human lung tissues, we measured viral copies generated at 1, 24, and 48 h postinfection. The data showed RNA levels increased for all viruses in the five lungs, although donor-donor variation was observed (see Fig. S1A in the supplemental material).

10.1128/mbio.03285-22.1 FIG S1 Replication dynamics of human, bat, or pangolin SARSr-CoVs in *ex vivo* human lung tissues and Calu-3 cells. (A) Human *ex vivo* lung tissues were infected with SARS-CoV-2, BtCoV-WIV1, or PCoV-GX (1 × 10^6^ TCID_50_/mL for all), and the culture supernatants were harvested at 1, 24, and 48 h postinfection. The virus RNA copies were measured using qRT-PCR. Results are shown as viral copies or changes compared to 1 h postinfection. (B) Calu-3 supernatants infected with BtCoV-WIV1 (MOI = 10), PCoV-GX (MOI = 10), or SARS-CoV-2 (MOI = 0.1) were harvested at 1 and 48 h postinfection, and viral replication was measured using qRT-PCR. Download FIG S1, PDF file, 0.5 MB. Copyright © 2023 Yang et al. 2023 Yang et al. https://creativecommons.org/licenses/by/4.0/ This content is distributed under the terms of the Creative Commons Attribution 4.0 International license.

To compare the replication dynamics of the three viruses, cultured human airway organoids were used. Airway organoids were cultured from the proximal ends of human lung tissues collected from a donor who underwent a surgical operation, a model that has previously been used to study SARS-CoV-2 infection (13). The data showed that viral antigens could be detected from the apical surface of the tracheal organoid for all three viruses, indicating a successful infection (Fig. 2C). When detecting the viral load in the supernatant, we found that SARS-CoV-2 demonstrated better replication efficiency than the other two viruses from 3 to 72 h postinfection. BtCoV-WIV1 showed a >10-fold-lower replication efficiency than SARS-CoV-2, whereas PCoV-GX did not replicate (Fig. 2D). Thus, PCoV-GX infection was abortive, and the increase in viral RNA may be related to the attached input viral particles.

### SARS-CoV-2 demonstrated higher replicative fitness than animal SARSr-CoVs in human nasal organoids.

**FIG 3:**
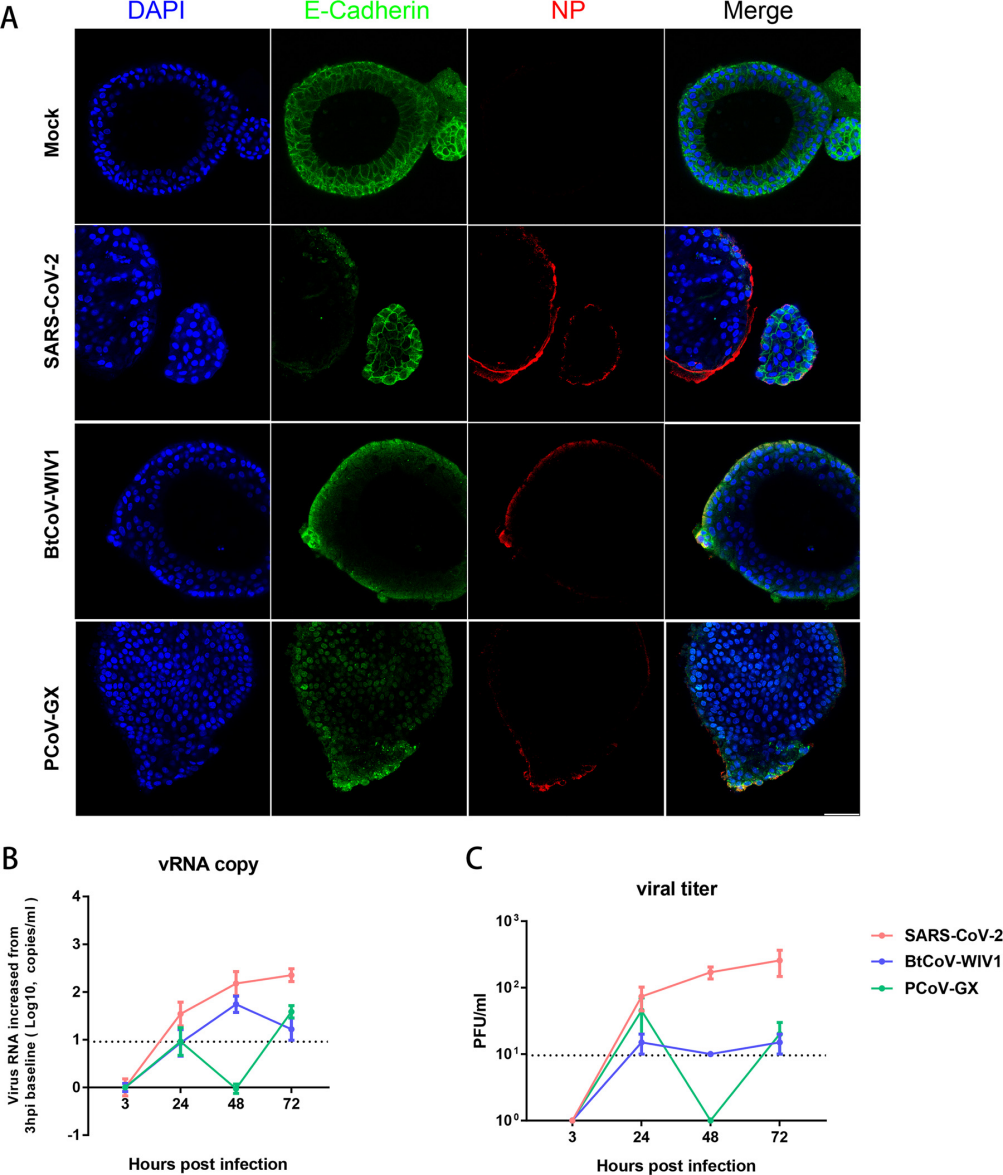
Infection and replication dynamics of human, bat, and pangolin SARSr-CoVs in human nasal organoids. (A) Human nasal organoids were infected with SARS-CoV-2, BtCoV-WIV1, or PCoV-GX. At 48 h postinfection, the tissues were fixed in 4% paraformaldehyde and immunohistochemically stained for the indicated cell markers: the viral NP protein (red), epithelial cell marker E-cadherin (green), and nuclear marker DAPI (blue). Mock-infected organoids were used as controls. Bars, 50 μm. (B and C) Culture supernatants were harvested at 3, 24, 48, and 72 h postinfection, and the qPCR-determined viral RNA copies (B) or infectious viral particles (in PFU per milliliter) (C) are shown. The detection limits are indicated.

As the primary target and entry portal of respiratory pathogens, nasal epithelial cells also express high levels of SARS-CoV-2 entry factors that favor robust SARS-CoV-2 replication, which constitutes the biological basis underlying viral pathogenesis and viral shedding (14). Thus, the measurement of viral replicative fitness in this organ could be a surrogate test for potential human spread. Human nasal organoids were prepared as previously reported (14). Next, we examined SARS-CoV-2, BtCoV-WIV1, and PCoV-GX replication kinetics in nasal organoids derived from healthy donors. The data revealed positive NP staining on the apical surfaces of all three viruses (Fig. 3A). Moreover, the determination of viral RNA or infectious particles revealed successful SARS-CoV-2 infection with an upward replication curve from 3, 24, 48, and 72 h postinfection. In contrast, neither BtCoV-WIV1 nor PCoV-GX replicated well in nasal organoids, as only a minimal level of infectious particles was produced (abortive infection) (Fig. 3B and C). Taken together, these results demonstrated that, compared with SARS-CoV-2, animal SARSr-CoVs exhibited less replicative fitness in the human nasal and airway environment.

### SARSr-CoVs from bats and pangolins hampered IFN induction and signaling.

To establish an environment for efficient replication upon infection, viruses normally encode proteins to suppress host innate immunity. For example, the pathogenicity of SARS-CoV-2 is partly attributed to its strong IFN-antagonizing activity, and several virus-encoded proteins impair type I IFN production or signaling pathways in human cells (15, 16). Therefore, we next determined whether animal SARSr-CoVs also inhibited human IFN pathways.

**FIG 4:**
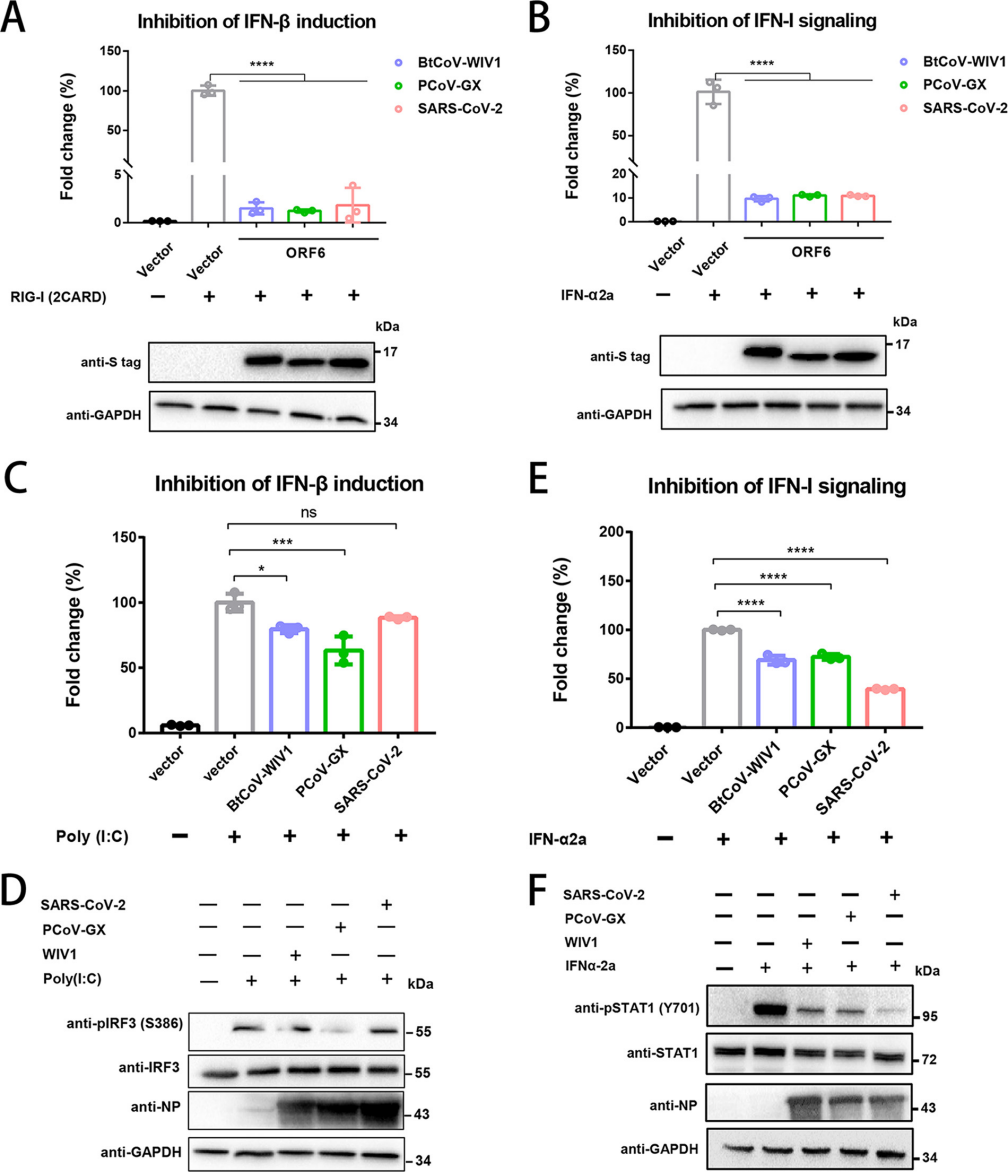
SARSr-CoVs hampered host type I interferon responses in humans. (A and B) ORF6 proteins from SARS-CoV-2, BtCoV-WIV1, or PCoV-GX blocked human type I interferon (IFN-I) production and signaling. Human HEK-293T cells were cotransfected with plasmids carrying IFN-β promoter, pRL-Tk, ORF6 gene, and RIG-I (2CARD) for IFN-I production assays (24 h) (A). HEK-293T cells were cotransfected with plasmids carrying ISRE-luc promoter, pRL-Tk, and ORF6 gene (16 h), followed by IFN-α2a stimulation (8 h) for IFN-I signaling assays (B). (C and D) Inhibition of IFN-β induction upon viral infection. Human HEK-293T-huACE2 cells were cotransfected with plasmids carrying IFN-β promoter and pRL-Tk (6 h), followed by viral infection (16 h) and poly(I·C) stimulation (24 h). The luciferase activity was measured (C). The same cells were also denatured in SDS loading buffer and analyzed with antibodies against total IRF3, phosphorylated IRF3 (phospho-IRF3-S386), viral NP, and GAPDH (D). (E and F) Inhibition of IFN-I signaling upon viral infection. Human HEK-293T-huACE2 cells were cotransfected with plasmids carrying ISRE-luc and pRL-Tk (6 h), followed by viral infection (16 h) and IFN-α2a stimulation (8 h). The luciferase activity was measured (E). The same cells were also denatured in SDS loading buffer and analyzed with antibodies against total STAT1, phosphorylated STAT1 (phospho-STAT1-Y701), viral NP, and GAPDH (F). Error bars are means ± standard deviations. Statistical significance was measured by one-way ANOVA with Dunnett’s correction. *, *P < *0.05; **, *P < *0.01; ***, *P < *0.001; ****, *P < *0.0001.

We first tested the ORF6 protein, which is a potent IFN-related antagonist of SARS-CoV-2 (15, 16). Using promoter luciferase assays, we showed that ORF6 proteins from SARS-CoV-2, BtCoV-WIV1, and PCoV-GX efficiently blocked either RIG-I-driven IFN-β production or IFN-α2A-driven IFN-stimulated response element (ISRE) signaling without showing significant differences, suggesting an immune escape function (Fig. 4A and B). Notably, most previous studies used viral proteins, which may not reflect the balance between virus replication-induced IFN activation and virus-encoded IFN antagonists during infection. Thus, we tested whether viral infection impaired host IFN-I activation. HEK293T-huACE2 cells were infected with SARSr-CoVs via an IFN-β promoter plasmid and poly(I·C) transfection. Luciferase signals were measured, and the results showed that BtCoV-WIV1 and PCoV-GX significantly suppressed IFN-β production, whereas SARS-CoV-2 showed weaker inhibitory activity, as measured in IFN-β promoter assays or IRF3 phosphorylation-blocking experiments (Fig. 4C and D). Notably, this assay reflected a balance between virus-encoded IFN antagonists and viral replication-induced IFN. Thus, the weaker inhibition by SARS-CoV-2 may have been related to the fact that the virus was also a stronger inducer of proinflammatory responses than the other two viruses, as demonstrated below. Similarly, all three viruses exhibited IFN-α2A-driven IFN-I signal-inhibitory activity, and SARS-CoV-2 was the most potent blocker, as shown in ISRE promoter assays and signal transducer and activator of transcription 1 (STAT1) phosphorylation experiments (Fig. 4E and F). Collectively, these findings demonstrated that both BtCoV-WIV1 and PCoV-GX hampered IFN production and signaling pathways.

### SARSr-CoVs from bats and pangolins induced weaker proinflammatory responses than SARS-CoV-2.

SARS-CoV-2 infection triggers imbalanced host responses, including dampened IFN activation and elevated proinflammatory responses, which contribute to severe disease in humans (17). Thus, efficient viral replication and elevated proinflammatory responses in respiratory or intestinal organs form the biological basis for respiratory viral pathogenesis and viral spread, as observed in SARS and influenza virus infections (11). To this end, we also compared the proinflammatory responses and induction of cell death in human lung organoids and cells between animal SARSr-CoVs and SARS-CoV-2 strains.

**FIG 5:**
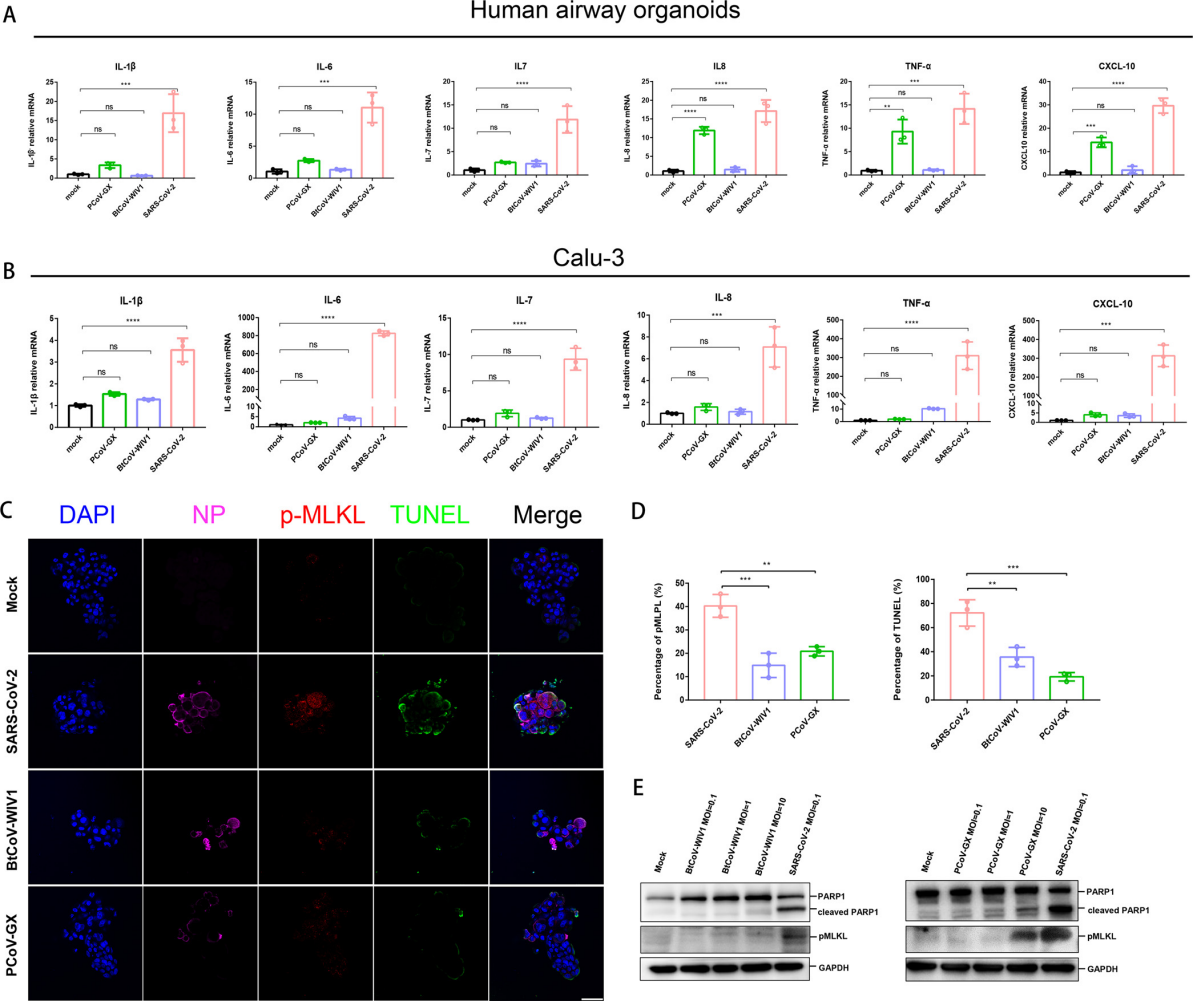
Bat and pangolin SARSr-CoVs induced weaker proinflammatory responses than SARS-CoV-2. (A and B) Viral infection induced proinflammatory responses in human airway organoids (A) and Calu-3 human lung cells (B). Human airway organoids or Calu-3 cells infected with BtCoV-WIV1, PCoV-GX, or SARS-CoV-2 were harvested at 48 h postinfection, and changes in the proinflammatory response were measured using qRT-PCR. (C to E) Viral infection induced necroptosis and apoptosis. Calu-3 cells were infected or mock infected with the indicated viruses, and the fixed cells were stained with antibodies indicating viral NP (pink), necroptosis (phosphorylated MLKL, p-MLKL, red), and apoptosis (TUNEL, green) (C). The percentages of p-MLKL-positive cells and TUNEL-positive cells were quantified (D). Calu-3 cells at 48 h postinfection were also harvested and subjected to Western blotting for PARP1 and cleaved PARP1 (an indicator of apoptosis), p-MLKL, or GAPDH (E). Mean values were analyzed using one-way ANOVA with Dunnett’s correction. *, *P < *0.05; **, *P < *0.01; ***, *P < *0.001; ****, *P < *0.0001; ns, not significant.

We first detected the expression of a series of signature cytokines and chemokines in human airway organoids and Calu-3 human lung cells. Owing to differences in replication efficiency, a higher viral dose was used for BtCoV-WIV1 and PCoV-GX (multiplicity of infection [MOI] = 10), and a lower dose was used for SARS-CoV-2 (MOI = 0.1) to test host responses in Calu-3 cells (Fig. S1B). Our results showed that in the two infection models, SARS-CoV-2 was the most potent inducer of proinflammatory cytokines (interleukin [IL]-1β, IL-6, IL-7, IL-8, tumor necrosis factor [TNF]-α, and C-X-C motif chemokine ligand-10 [CXCL-10]), which play critical roles in immune dysregulation in patients with CoV disease 2019 (COVID-19) (17). In contrast, BtCoV-WIV1 infections did not induce any of these responses in the two models, whereas PCoV-GX only induced IL-8, TNF-α, and CXCL-10 in airway organoids (Fig. 5A and B).

Next, we assessed the activation of apoptotic and necroptotic cell death pathways, which lead to pronounced inflammatory responses upon SARS-CoV-2 infection (18). Necroptosis activation was detected by analyzing the phosphorylation of mixed-lineage kinase domain-like pseudokinase (MLKL), and apoptosis was detected using terminal deoxynucleotidyl transferase dUTP nick end labeling (TUNEL) staining and poly(ADP-ribose) polymerase 1 (PARP1) cleavage assays (18). The data showed that SARS-CoV-2 infection triggered prominent phospho-MLKL activation (40%), TUNEL staining (70%), and PARP1 cleavage. In contrast, both BtCoV-WIV1 and PCoV-GX showed limited phospho-MLKL activation (<20%), low TUNEL staining (<40%), and weak PARP1 cleavage (Fig. 5C to E). These data were consistent with the cytokine expression profiles, which showed that SARS-CoV-2 was the most potent inducer among the three, whereas neither BtCoV-WIV1 nor PCoV-GX induced pronounced inflammation in human airway or lung cells. Thus, animal SARSr-CoVs may not be able to cause severe disease or spread though airway as efficiently as SARS-CoV-2.

### Adaptive immune barriers against SARS-CoV-2 were shown to be less potent against SARSr-CoVs from bats and pangolins.

**FIG 6:**
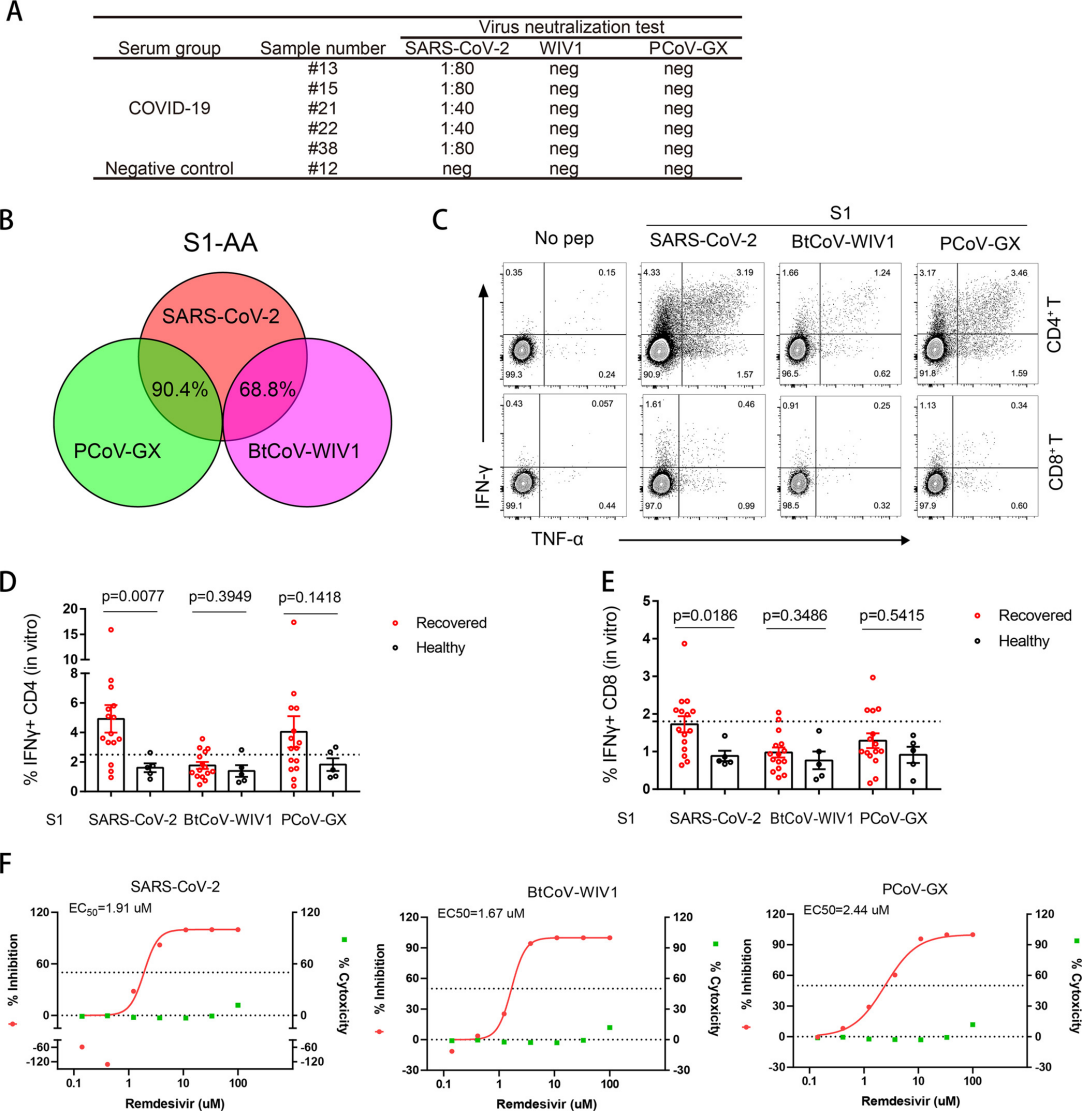
Bat and pangolin SARSr-CoVs partially escaped adaptive immune barriers against SARS-CoV-2. (A) Serum samples from COVID-19 convalescents were used to test their neutralizing activities against SARS-CoV-2, BtCoV-WIV1, and PCoV-GX. (B) Sequence identities of S1 peptides from SARS-CoV-2, BtCoV-WIV1, and PCoV-GX, which were used for PBMC stimulation. (C to E) PBMCs collected from 15 COVID-19 convalescents were stimulated with S1 peptide pools in the presence of 20 U/mL recombinant IL-2 and 20 ng/mL recombinant IL-7 for 10 days. The frequencies of IFN-γ and TNF-α double-positive CD4^+^ T cells or CD8^+^ T cells from one donor are shown, as determined by flow cytometry analysis (C). The frequencies of IFN-γ single-positive CD4^+^ T cells (D) and CD8^+^ T cells (E) for all 15 donors were also calculated. The threshold was based on the healthy donor group + 2 standard deviations, shown as dashed lines. Mann-Whitney tests were used to determine statistical significance, and *P* values are shown. (F) Antivirus efficiency of remdesivir against the three viruses. Vero E6 cells were challenged with the three viruses at an MOI of 0.05, and the inhibition or cytotoxicity ratios were measured after incubation in the presence of remdesivir for 24 h.

Owing to the wide prevalence of adaptive immune barriers against SARS-CoV-2, we tested whether SARSr-CoVs from bats and pangolins escaped these barriers. First, we used convalescent-phase sera collected from patients with COVID-19. Our data showed that none of the five serum samples neutralized BtCoV-WIV1 or PCoV-GX, although they showed variable activity against SARS-CoV-2 (Fig. 6A). The memory T-cell response is important for limiting human diseases caused by virus reinfection. We therefore tested whether the memory T-cell responses to SARS-CoV-2 infection were responsive to BtCoV-WIV1 and PCoV-GX. Fifteen peripheral blood mononuclear cell (PBMC) samples collected from convalescent patients with COVID-19 (infected in early 2020) were isolated and incubated *in vitro* with 10 μM S1 peptide pool for 10 days, followed by analysis of memory T-cell responses. Notably, 90.4% of the PCoV-GX S1 peptides were identical to SARS-CoV-2 in sequence, compared with 68.8% of the BtCoV-WIV1 S1 peptides to SARS-CoV-2 (Fig. 6B). Correspondingly, the frequencies of IFN-γ^+^ CD4^+^ or IFN-γ^+^ CD8^+^ T-cell responses were the highest following SARS-CoV-2 S1 peptide stimulation, and moderate responses were observed against PCoV-GX. T-cell responses against BtCoV-WIV1 were minimal (Fig. 6C to E and Fig. S2). Although a higher response was expected when using peptides against more conserved proteins (e.g., NP), the absent or weak memory T-cell response against S1 should dampen the overall efficacy of SARS-CoV-2-based T- or B-cell memory responses against BtCoV-WIV1 and PCoV-GX infections. Finally, we tested the antiviral effects of remdesivir, a broad-spectrum antiviral nucleotide analog that has also been used against SARS-CoV-2 (19). Our data showed that remdesivir was effective against all three viruses at a nearly identical half-maximal effective concentration, suggesting that the drug target was conserved among SARSr-CoVs (Fig. 6F). Collectively, these findings suggested that SARSr-CoVs from bats and pangolins partially escaped the adaptive immune barriers to SARS-CoV-2.

10.1128/mbio.03285-22.2 FIG S2 Gating strategy for activated memory T-cell detection in COVID-19 convalescents. PBMCs from donors were expanded for 10 days, followed by S1 peptide pool stimulation. Surface and intracellular immunostaining were used to identify IFN-γ- or TNF-α-positive CD4 or CD8 subsets. Download FIG S2, PDF file, 0.5 MB. Copyright © 2023 Yang et al. 2023 Yang et al. https://creativecommons.org/licenses/by/4.0/ This content is distributed under the terms of the Creative Commons Attribution 4.0 International license.

## DISCUSSION

In this study, we established an integrated framework to assess the zoonotic potential of bat and pangolin CoVs. Using human *ex vivo* lung tissues, human airway and nasal organoids, and human lung cells, we compared viral cell tropism, replication dynamics, and how the virus interacted with human immune barriers, including IFN signaling, inflammatory pathways, and adaptive immune barriers to SARSr-CoVs from humans, bats, and pangolins. Our data indicated that bat or pangolin SARSr-CoVs showed less replicative fitness in human airway cells or nasal organoids and triggered weaker proinflammatory responses than did SARS-CoV-2. Collectively, these animal viruses do not fully adapt to spread or cause severe diseases, which could result in successful zoonoses in humans. We believe that this experimental framework provides a pathway for identifying animal CoVs with the potential to cause zoonotic events that cannot be predicted from viral sequence information alone.

Our results suggested that direct bat CoV zoonotic transmission to humans is very difficult. SARS-CoV-2 interacts with hundreds of human proteins during infection in human cells, and these proteins are involved in cell entry, host innate immunity, and proviral factor expression (20). Thus, it is conceivable that a bat CoV may interact with hundreds of bat proteins for successful infection and transmission, while these proteins likely differ from their human counterparts owing to the phylogenetic positions of bats and humans (21). Subsequently, bat CoVs that break through these three bottlenecks are extremely rare. For example, very few animal SARSr-CoVs use human ACE2, and even fewer can be isolated (3, 10). Our data showed that even isolated viruses require further adaptation for efficient replication in humans. Thus, risk assessment for SARSr-CoVs should not be purely based on viral genetic analysis, and an integrated framework that includes genetic, virological, and ecological analyses should be applied for future zoonosis preparedness.

Our data also revealed the possible threats posed by animal SARSr-CoVs. Notably, if animal SARSr-CoVs break through the first bottleneck that allows cell entry, these SARSr-CoVs may subvert human innate immunity and experience replication adaptation in human cells. As shown by our data and previous studies, both BtCoV-WIV1 and PCoV-GX may infect human nasal or airway epithelial cells, albeit at a lower efficiency than SARS-CoV-2 (6, 22, 23). These facts suggest that the host proteins required for SARSr-CoVs to escape host innate immunity and to replicate are somehow similar between bats or pangolins versus humans, although the efficiencies may differ. For example, IFN antagonist activity is conserved for ORF6 among SARSr-CoVs (24); however, whether the proteins supporting SARSr-CoV replication are similar between bats and humans is still unclear. Future study is needed using CRISPR knockout libraries against humans, bats, and pangolins. Moreover, our data also showed that some of the currently available therapeutic methods, such as neutralizing antibodies, may not be fully protective against bat and pangolin SARSr-CoVs and that additional specific or pan-Sarbecovirus therapeutics should be developed. Finally, mammalian game animals and wild or semiwild animals that are commonly traded (e.g., civets and pangolins) may provide links between bats and humans, because these animals often have close contact with both humans and other wildlife species (25). Therefore, both ecological efforts, including stopping deforestation and wildlife trade, and investigation of viruses carried by game animals should be undertaken.

Based on these collective findings, we have proposed an integrated framework to determine the zoonotic risk of CoVs carried by bats or other wildlife animals. Using this framework, we have demonstrated that BtCoV-WIV1 and PCoV-GX have low probability of causing human zoonoses. Our work provides important conceptual and experimental information to improve our preparedness against future CoV diseases.

## MATERIALS AND METHODS

### Viruses.

SARS-CoV-2 WIV04 (GenBank accession number MN996528.1) was isolated from the bronchoalveolar lavage fluid of patients, as described previously (4). BatCoV-WIV1 was isolated from bat fecal samples (GenBank accession number KF367457.1) (8). Malayan pangolin CoVs (PCoV-GX) were isolated from smuggled pangolins (GenBank accession number MT072864.1) (7). SARS-CoV-2, BtCoV-WIV1, and PCoV-GX were propagated and titrated in VeroE6 cells. According to regulations from the biosecurity assessment committee at WIV, SARS-CoV-2 was cultured in a biosafety level 3 (BSL3) environment, whereas BtCoV-WIV1 and PCoV-GX were cultured in a BSL2 environment.

### Cell lines.

Calu-3 cells were cultured in minimum Eagle’s medium (MEM; Gibco) containing 10% fetal bovine serum (Gibco), 1% MEM nonessential amino acids (Gibco), and 1% sodium pyruvate (100 mM; Gibco). VeroE6 and HEK293T-huACE2 cells were grown and propagated in Dulbecco’s modified Eagle’s medium (Gibco) supplemented with 10% fetal bovine serum. Cells were grown at 37°C in a humidified atmosphere containing 5% CO_2_.

### Plasmids, antibodies, and reagents.

The overexpression plasmid pCMV-RIG-I-FLAG (2CARD) and reporter plasmids pIFN-luc, pISRE-luc, and pRluc-TK were used, as described previously (16). Viral ORF6 proteins were cloned into the pCAGGS-S-tag vector using multiple cloning sites.

Rabbit anti-CD68 (catalog number 76437T; 1:500), anti-phospho-IRF3 (S386; catalog number 37829S; 1:1,000), anti-PARP1 (catalog number 9542S; 1:1,000), and anti-phospho-MLKL (S358; (catalog number 91689S; 1:500) antibodies were purchased from Cell Signaling Technology (Danvers, MA, USA). Recombinant antibodies, including anti-podoplanin (catalog number ab236529; 1:2,000), were purchased from Abcam (Cambridge, United Kingdom). Rabbit anti-TTF1 (catalog number A3292; 1:100), anti-IRF3 (catalog number A1118; 1:5,000), anti-STAT1 (catalog number A19563; 1:1,000), and anti-phospho-STAT1 (Y701; catalog number AP0054; 1:1,000) antibodies were purchased from ABclonal. Mouse anti-E-cadherin (catalog number 610181, 1:50) was acquired from BD Biosciences. Rabbit anti-α-tubulin (catalog number 11224-1-AP; 1:4,000) and mouse mouse-anti-glyceraldehyde 3-phosphate dehydrogenase (GAPDH; catalog number 60004-1; 1:5,000) were acquired from Proteintech. Rabbit anti-SARS-CoV-2-NP antibodies were prepared in-house. Allophycocyanin (APC)/Cy7 anti-human CD3 (catalog number 300426; 1:100), fluorescein isothiocyanate (FITC) anti-human CD4 (catalog number 300506; 1:100), Pacific blue anti-human CD8 (catalog number 301033; 1:100), phycoerythrin anti-human TNF-α (catalog number 502909; 1:200), and APC anti-human IFN-γ (catalog number 506510; 1:200) were purchased from Biolegend (CA, USA). Poly(I·C) (tlrl-pic) was obtained from Invitrogen (Carlsbad, CA, USA), and recombinant human IFN-α2a (catalog number P5646) was purchased from Beyotime. Remdesivir (catalog number GS-5734) was obtained from MedChemExpress.

### Culture of human *ex vivo* lung tissues.

Fresh human lung tissues were obtained from patients undergoing surgery at Tongji Hospital (Wuhan, China). Human blood samples, either from COVID-19 convalescents or healthy donors, were also obtained from Tongji Hospital. Human lung tissues were processed into small pieces. Fragments of human lung tissues were infected with each virus at 1 × 10^6^ 50% tissue culture infective dose (TCID_50_)/mL. The virus inoculum was removed 1 h after attachment. The tissues were washed three times with phosphate-buffered saline (PBS) and placed directly into 24-well plates with 1.2 mL F12K culture medium containing 100 U/mL penicillin and 100 U/mL streptomycin in a 37°C incubator with an atmosphere containing 5% CO_2_. Supernatants were collected at 1, 24, and 48 h postinfection for quantitative reverse transcription-PCR (qRT-PCR) analysis. Lung tissue was harvested and fixed at 24 h postinfection in 4% paraformaldehyde for immunohistochemical staining.

### Ethics statement.

The ethics committees of designated hospitals for emerging infectious diseases approved all human materials.

### Human airway and nasal organoids infection.

The establishment, maintenance, and differentiation of human airway and nasal organoids were based on previous reports (13, 14). For infection, organoids were digested and seeded in 24-well plates and inoculated at an MOI of 0.1 for SARS-CoV-2, BtCoV-WIV1, and PCoV-GX. The infected samples were harvested at 3, 24, 48, and 72h and analyzed for viral titer and cytokine expression. Briefly, the titers of infectious virus were measured using a plaque assay. Vero E6 cells were seeded at 1.8 × 10^5^ cells per well in a 48-well plate and inoculated with serially diluted samples for 1 h, then cells were overlaid with 0.8% cellulose semisolid medium supplemented with 2% fetal bovine serum, and after 4 to 5 days, the plaques were calculated using a microscope. Organoid RNA extractions were performed using an RNAprep pure cell/bacteria kit (Tiangen), cDNA was synthesized with a HiScript II 1st strand cDNA synthesis kit, and qRT-PCR was performed with *Taq* Pro Universal SYBR qPCR master mix (Vazyme, China). For the immunostaining assay, organoids were challenged with a higher viral titer (MOI = 1) and fixed at 72h postinfection, and the immunofluorescence was determined with the antibodies mentioned above and analyzed using confocal microscope (Leica Stellaris 8).

### Immunohistochemistry.

Human lung tissues fixed in 4% paraformaldehyde were embedded in paraffin and sectioned at 4-μm thickness with a Thermo Fisher Scientific HM 355S rotary microtome. Deparaffinization and antigen retrieval were performed prior to immunofluorescence staining. Briefly, tissue sections were incubated with 0.1% Sudan Black B solution and blocked with 1× PBS containing 5% bovine serum albumin and 0.1% Triton X-100. Polyclonal rabbit primary antibodies were raised against the SARS-CoV-2 N glycoprotein (1:800), which cross-reacted with BtCoV-WIV1 and PCoV-GX. Anti-CD68 antibodies were used for macrophages, anti-podoplanin antibodies were used for type I pneumocytes, and anti-TTF1 antibodies were used for type II pneumocytes. FITC-conjugated Affinipure goat anti-rabbit IgG (H+L) (1:100) and Cy3-conjugated Affinipure goat anti-rabbit IgG (H+L) (1:100) were used. The stained slices were imaged using a Panoramic MIDI system (3DHISTECH).

### Immunofluorescence.

Calu-3 cells were infected for 48 h at MOIs of 0.1 for SARS-CoV-2 and 10 for BtCoV-WIV1 and PCoV-GX. Cells were then fixed, and apoptosis assays were performed using a TUNEL assay kit purchased from Thermo Fisher (catalog number C10617) according to the manufacturer’s protocols. Anti-phospho-MLPL (catalog number S358, Abcam) was selected as a marker for necroptosis analysis, and rabbit anti-NP and 4′,6-diamidino-2-phenylindole (DAPI) were used to stain the virus and nuclei, respectively. Images were obtained using a confocal microscope (Andor Dragonfly 202).

### RNA extraction and qRT-PCR analysis.

The viral RNA in supernatants collected at different time points was extracted using a QIAamp viral RNA minikit (Qiagen, Valencia, CA, USA) and quantified using a One-Step qRT-PCR SYBR green kit (Vazyme, China) with standard curves. For cytokine quantification, cDNA was synthesized using a HiScript II 1st Strand cDNA synthesis kit, and qRT-PCR was performed using *Taq* Pro Universal SYBR qPCR Master Mix (Vazyme).

### IFN-I production and signaling luciferase assays.

IFN-I inhibition assays of virus ORF6 proteins were performed as previously described (16, 24). For IFN-I production assays, HEK293T-hACE2 cells were transfected with an optimized plasmid (90 ng IFN-β-luc, 10 ng pRL-Tk) using Lipo3000 transfection reagent (Invitrogen), according to the manufacturer’s instructions. Viral infection was performed 6 h later. Cells were treated with poly(I·C) after 16 h and lysed by passive lysis after an additional 24 h. Luciferase activity was measured using a kit (Promega, Madison, WI, USA) according to the manufacturer’s instructions. For IFN-I signaling, HEK293T-hACE2 cells were cotransfected with 90 ng ISRE-luc and10 ng pRL-Tk; after incubation for 6 h, viral infection was performed, and 1,000 U/mL IFN-α2a was added to stimulate transfected cells after an additional 16 h. After another 8 h of incubation, luciferase activity was determined using a kit (Promega) according to the manufacturer’s guidelines and measured on a luminometer (Infinite M200 pro, Tecan).

### Western blot analysis.

Cells infected with the indicated viruses were lysed using RIPA lysis buffer supplemented with 1% phenylmethylsulfonyl fluoride (Beyotime, China). The cell lysates were mixed with SDS loading buffer and denatured in boiling water for 10 min. Equal numbers of samples were subjected to SDS-polyacrylamide gel electrophoresis and transferred onto polyvinylidene difluoride membranes. After blocking, the membranes were probed with the following primary antibodies: anti-IRF3, anti-phospho-IRF3, anti-STAT1, anti-phospho-STAT1 (Y701), anti-GAPDH, and anti-SARS-CoV-2-NP (1:2,000). Horseradish peroxidase-conjugated secondary antibodies (1:5,000) were added for 1 h, and the separated proteins were detected with chemiluminescent horseradish peroxidase substrate (Millipore) using a ChemiDoc imaging system (Bio-Rad Laboratories, Hercules, CA, USA).

### Virus titer neutralization assay.

Serum samples (50 μL) were diluted 2-fold, starting at 1:20, and preincubated with a diluted virus (4 × 10^3^ TCID_50_/mL) at 37°C for 30 min. The mixture was then added to Vero-E6 cells preseeded in 96-well plates and incubated at 37°C for 1 h. The mixture was then replaced with fresh culture medium. The results were read after 4 to 5 days of incubation. The highest serum dilution that completely inhibited the cytopathic effect was defined as the neutralization antibody titer.

### Remdesivir antiviral activity assay *in vitro*.

To measure the antiviral effects of remdesivir, we performed efficacy and cytotoxicity assays using Vero E6 cells. Remdesivir was first diluted in dimethyl sulfoxide to obtain a 10 mM solution; concentrations of 0.14 to 100 μM were used in this assay. Cells were plated at 3 × 10^4^ cells/well and infected with SARS-CoV-2, BtCoV-WIV1, or PCoV-GX at an MOI of 0.05. Viruses were removed after 1 h of incubation, and the cells were washed with PBS. Fresh medium containing a series of dilutions of remdesivir was added. After 24 h, the supernatants were harvested, and virus quantification was determined by RT-PCR.

### PBMC isolation and stimulation *in vitro*.

PBMCs from patients with COVID-19 were isolated by density gradient centrifugation using Ficoll-Paque (catalog number 17-5442-02, GE Healthcare) according to the manufacturer’s guidelines. Briefly, (5 to 8) × 10^5^ cells were cultured for 10 days in medium containing 20 U/mL recombinant IL-2, 20 ng/mL recombinant IL-7, and 10 μM S1 peptide. During the culture period, half of the medium was replaced every 3 days. On day 10, the cells were stimulated with medium supplemented with the peptide pool and GolgiPlug (BD Biosciences, San Diego, CA) overnight at 37°C in an atmosphere containing 5% CO_2_.

### Flow cytometry.

PBMCs stimulated overnight from day 10 were harvested and washed for staining assays. First, cells were incubated with reagents from a live/dead fixable viability kit (catalog number 423102, BioLegend) at room temperature in the dark for 15 min. The cells were then washed again, and surface staining was performed with the following antibodies: APC/Cy7 anti-human CD3, FITC anti-human CD4, and Pacific blue anti-human CD8. Cells were subsequently fixed and permeabilized using a Cytofix and Perm kit (BD Bioscience) and stained with the following intracellular cytokine antibodies: phycoerythrin (PE) anti-human TNF-α and APC anti-human IFN-γ. Finally, the cells were resuspended in 200 μL fluorescence-activated cell soring buffer and analyzed on a BD FACSAria III instrument. Data were evaluated using FlowJo software.

### Statistical analysis.

Statistical variations were measured using one-way analysis of variance (ANOVA) or Mann-Whitney tests, as indicated in the figure legends, with GraphPad Prism version 7. Data presented are means ± standard deviations (*, *P < *0.05; **, *P < *0.01; ***, *P < *0.001; ****, *P < *0.0001; ns, not significant).
