# Supplementary figures and images for: Strategy To Assess Zoonotic Potential Reveals Low Risk Posed by SARS-Related Coronaviruses from Bat and Pangolin
Source: mBio. 2023 Feb 14;14(2):e03285-22. doi: 10.1128/mbio.03285-22 (PMC10127581; doi:10.1128/mbio.03285-22)

**A**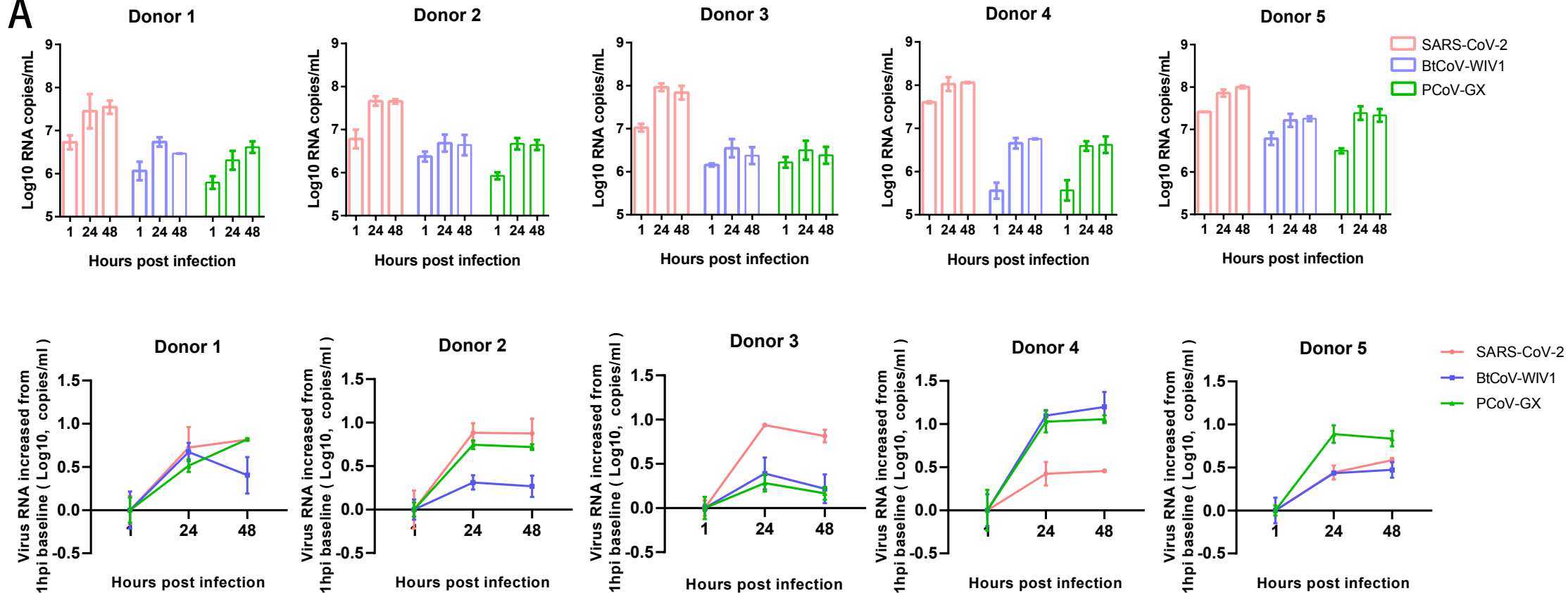**B**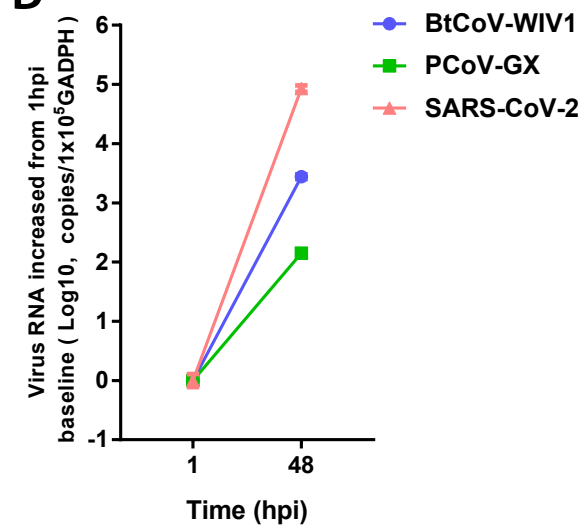

Supplement: FIG S1 [file mbio.03285-22-s0001.pdf]

A

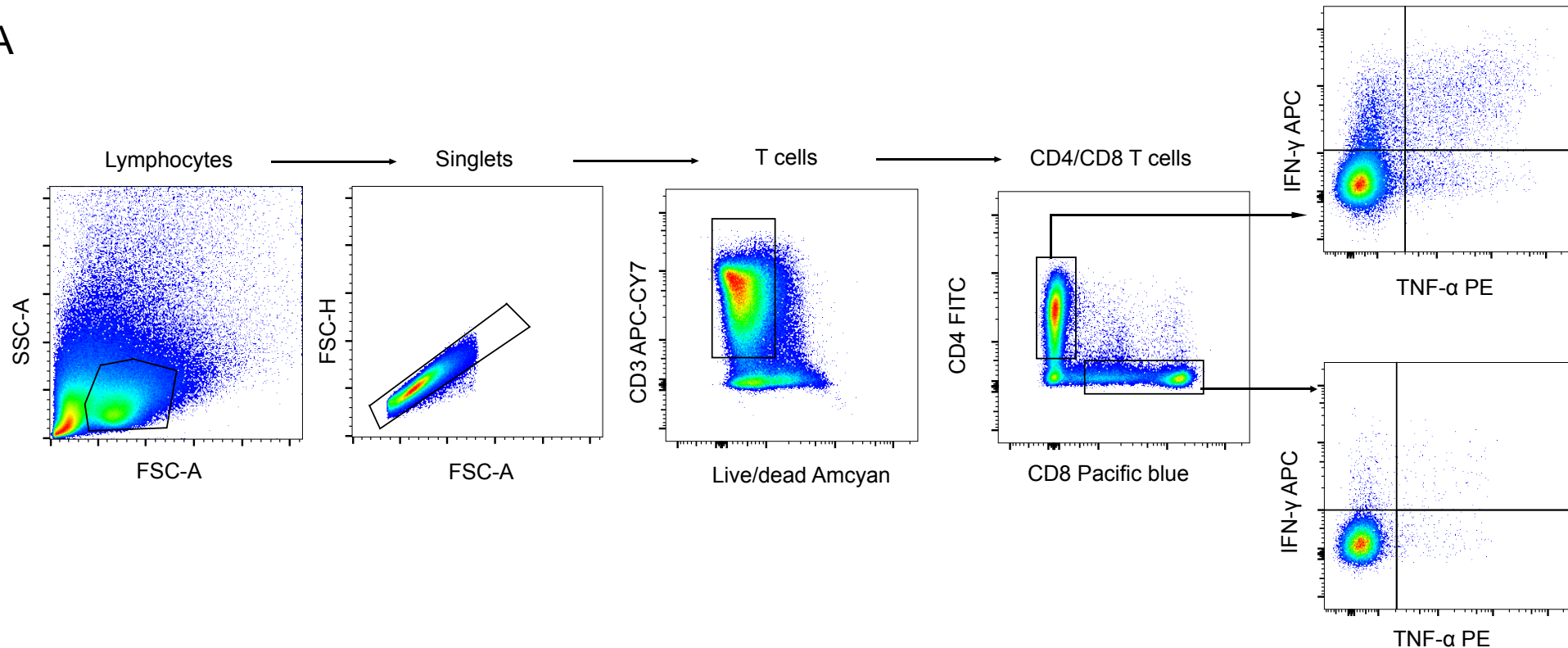

Supplement: FIG S2 [file mbio.03285-22-s0002.pdf]
